# High performance transcription factor-DNA docking with GPU computing

**DOI:** 10.1186/1477-5956-10-S1-S17

**Published:** 2012-06-21

**Authors:** Jiadong Wu, Bo Hong, Takako Takeda, Jun-tao Guo

**Affiliations:** 1 School of Electrical and Computer Engineering, Georgia Institute of Technology, Atlanta, Georgia, 30332, USA; 2 Department of Bioinformatics and Genomics, College of Computing and Informatics, University of North Carolina at Charlotte, Charlotte, North Carolina 28223, USA

## Abstract

**Background:**

Protein-DNA docking is a very challenging problem in structural bioinformatics and has important implications in a number of applications, such as structure-based prediction of transcription factor binding sites and rational drug design. Protein-DNA docking is very computational demanding due to the high cost of energy calculation and the statistical nature of conformational sampling algorithms. More importantly, experiments show that the docking quality depends on the coverage of the conformational sampling space. It is therefore desirable to accelerate the computation of the docking algorithm, not only to reduce computing time, but also to improve docking quality.

**Methods:**

In an attempt to accelerate the sampling process and to improve the docking performance, we developed a graphics processing unit (GPU)-based protein-DNA docking algorithm. The algorithm employs a potential-based energy function to describe the binding affinity of a protein-DNA pair, and integrates Monte-Carlo simulation and a simulated annealing method to search through the conformational space. Algorithmic techniques were developed to improve the computation efficiency and scalability on GPU-based high performance computing systems.

**Results:**

The effectiveness of our approach is tested on a non-redundant set of 75 TF-DNA complexes and a newly developed TF-DNA docking benchmark. We demonstrated that the GPU-based docking algorithm can significantly accelerate the simulation process and thereby improving the chance of finding near-native TF-DNA complex structures. This study also suggests that further improvement in protein-DNA docking research would require efforts from two integral aspects: improvement in computation efficiency and energy function design.

**Conclusions:**

We present a high performance computing approach for improving the prediction accuracy of protein-DNA docking. The GPU-based docking algorithm accelerates the search of the conformational space and thus increases the chance of finding more near-native structures. To the best of our knowledge, this is the first *ad hoc *effort of applying GPU or GPU clusters to the protein-DNA docking problem.

## Background

Protein-DNA interactions play crucial roles in many key biological processes. One of these processes is transcriptional regulation, in which transcription factors (TFs) bind to specific DNA binding sequences to either activate or repress the expression of their regulated genes. Transcription factors form a distinct group of DNA binding proteins in terms of function and binding specificity [1]. Owning to their roles in cancer development, transcription factors are potential drug targets for cancer therapy [2-4]. Therefore, knowledge of transcription factor-DNA interaction at a structural level not only can help us better understand the protein-DNA recognition and binding specificity, more importantly it can also offer guidance in targeted drug design. Moreover, structure-based transcription factor binding site prediction at genome scale has received much deserved attention recently because structure-based approaches have the advantage to consider both the position interdependence of TFs and the contribution of DNA flanking sequences in assessing TF-DNA binding specificity [5-9]. Despite rapid technological advances in experimental structure determination, only a small percentage of the structures in Protein Data Bank (PDB) are TF-DNA complexes [10]. Computational modeling, on the other hand, provides a cost-efficient alternative to the usually time-consuming experimental methods. Previous studies have demonstrated that molecular docking can obtain accurate complex structures for protein-protein, protein-peptide, and protein-ligand interactions. However, protein-DNA docking still lags behind due to our limited knowledge of protein-DNA interactions and it remains one of the challenging problems in the field of structural bioinformatics.

There are two key issues in general protein-DNA docking. One is a potential function for accurate evaluation of protein-DNA binding affinity. The other is conformational sampling of the complex structures. It should be noted that these two issues are related since the sampling methods generally need the energy function to guide the search. Improvement has been made in the development of knowledge-based potentials for assessing the binding affinity between protein and DNA. These knowledge-based potentials are developed based on the mean-force theory and are more attractive due to their relative simplicity and ease of use. These potentials generally vary in their resolution levels, from residue-based to atom-based potentials and in their distance scales, from distance-independent to distance-dependent [9,11,12]. Studies have shown that specific interaction environments around the contacting amino acids and nucleotides contribute significantly to the binding affinity [13,14]. To take structural context into consideration, Liu et. al. have previously developed a knowledge-based, multi-body interaction potential for protein-DNA interaction [11]. This statistical potential can describe the effects of DNA structure deformation and local interaction environments. By using three structurally adjacent nucleotides (termed DNA tri-nucleotides or triplets) as the basic interaction units, we developed a rigid-body docking algorithm using Monte-Carlo simulations and further extended the rigid-body docking algorithm to a semi-flexible docking approach in case the DNA structure is unknown [8]. In semi-flexible docking, we use a number of representative DNA structures that are generated by clustering DNA structures from solved protein-DNA complexes. Each DNA structure model is docked with the target protein structure using the rigid-body docking algorithm. The best conformation from all the docked protein-DNA combinations is selected as the final complex model.

We have demonstrated the effectiveness of our protein-DNA docking algorithm and its application in structure-based transcription factor binding site prediction [8]. However, the docking algorithm is very computation expensive. For example, our semi-flexible experiments with 45 TF-DNA complexes (200 Monte-Carlo simulation runs for each complex) needed 130, 000 CPU hours when executed on 2.8GHz Intel Xeon processors (3 weeks on a 240-node CPU cluster). More importantly, the performance of the docking algorithm depends substantially on the coverage of the search space. It has been mathematically proved that for any random search algorithm to find a global optimal solution, the probability of repeatedly missing any measurable subsets of the search space must be zero [15]. This effectively requires our Monte-Carlo simulation procedure to search over the entire solution space, which is infeasible and can only be approximated by increasing the number of random samples. The computation cost therefore hinders the implementation and execution of our algorithm, especially for parameter optimization and large-scale testing runs. In this paper, our objective is to improve conformational search for TF-DNA docking algorithm through Graphics Processing Unit (GPU) computing. GPU has recently evolved from a fixed-function graphical device into a highly programmable parallel processor, and has been successfully deployed to accelerate a broad range of scientific applications [16,17]. GPUs support massive thread-level parallelism and can significantly accelerate parallel workloads. But the architectural features of GPUs also pose unique challenges to GPU application design, especially on the patterns of their memory accesses and execution paths. In this paper, we present the GPU implementation of our protein-DNA docking algorithm, which carefully manages memory accesses and execution paths to explore GPU acceleration. We further scale our algorithm from a single GPU card to a cluster of GPUs and achieve significant performance improvement. Although the application of GPU to other docking problems have been previously investigated [18], to the best of our knowledge, this is the first *ad hoc *effort of applying GPU or GPU clusters to the protein-DNA docking problem.

The effectiveness of our new method is validated through extensive experiments. Our GPU algorithm exhibits a 28x speedup on an individual Nvidia M2070 GPU over a single 2.8GHz Xeon core, and achieves a sustained performance of 10.4 TFLOPS using a cluster of 128 GPUs, which equals the capability of a conventional cluster with 3600 CPU cores. With the benefit of such improved computation capability, we are able to increase the number of random samples for the Monte-Carlo simulation procedure and thus expand the traversal in the search space. Experimental results show that GPU acceleration leads to higher rate of successful prediction of TF-DNA complexes. We also tested our GPU method on the rigid TF-DNA docking benchmark with carefully selected 38 cases [19]. We found that more random sampling can improve the performance of the easy cases but has almost no effect on the hard cases, suggesting that better energy functions and search algorithms are needed for these hard cases.

## Methods

### Datasets

A non-redundant set of 75 TF-DNA complex structures (less than 35% protein sequence identity) was generated from PDB [10]. Each of these TF-DNA complex structures was solved by X-ray crystallography with a resolution of 3.0 Å or better. Since transcription factors are not well annotated in PDB, we developed an in-house program for automatic identification of TF-DNA complexes by combing information from Gene Ontology (GO) terms [20], UniProt keywords [21], and PDB keywords. Another dataset used for testing our GPU algorithm is the rigid TF-DNA docking benchmark [19]. This benchmark contains 38 non-redundant cases that are classified into two groups in terms of expected docking difficulty. Each case in the benchmark is a TF-DNA binding unit, which is defined as an entity of a DNA double-helix and one or more TF-chains that interact with each other with at least three residue-residue contacts based on a heavy-atom distance cutoff of 4.5 Å. The degree of difficulty is assigned based on the strength of TF-DNA interactions in terms of the number of residue-base contacts (NRBCs) [19]. The easy group has 21 cases and the hard group has 17 cases.

### TF-DNA docking algorithm

**Figure 1:**
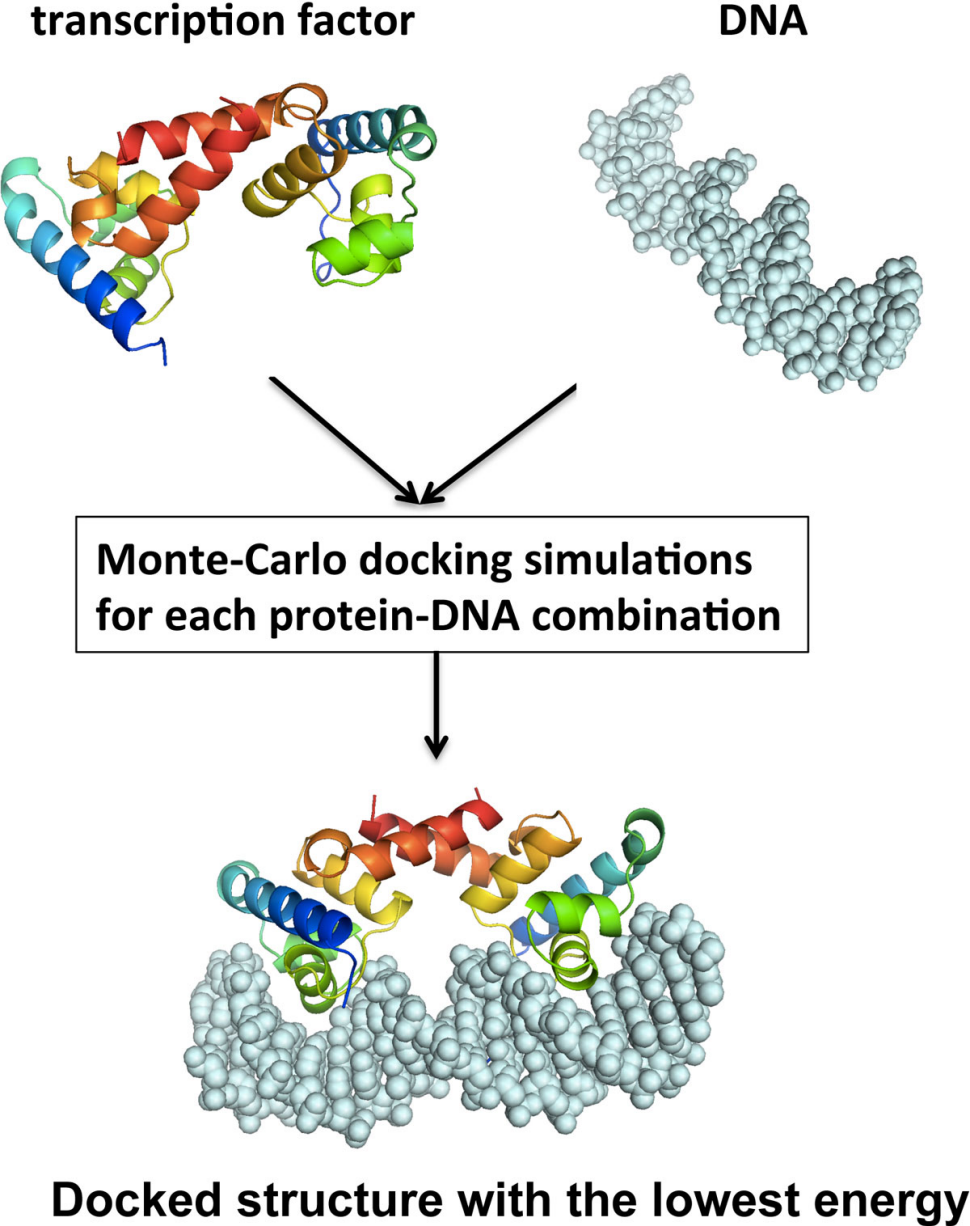
**Schematic representation of the docking procedure**.

Figure 1 shows the overall framework of our rigid-body docking algorithm as described previously [8]. Briefly, for a given pair of transcription factor and DNA, our algorithm searches for a docked TF-DNA structure that has the lowest interaction energy using a Monte-Carlo simulated annealing approach. Each iteration of the Monte-Carlo simulation consists of two steps: docking energy calculation and conformational sampling. The energy function includes the protein-DNA binding affinity (*E_binding_*), the atomic van der Waals (VDW) packing energy (*E_packing_*), and the constraint energy (*E_constraint_*) as seen in Equation 1 (*W_packing _*and *W_constraint _*are weight factors).

(1) $$E=E_{binding}+W_{packing}\;E_{packing}+W_{constraint}\;E_{constraint}$$

The protein-DNA binding affinity, which dominates the docking energy, is evaluated using a knowledge-based distance-dependent amino acid-nucleotide interaction potential [11]. The binding affinity between a DNA triplet and a protein residue is determined by three factors: 1) the composition of the DNA triplet; 2) the type of the protein residue; and 3) the distance between the triplet and the residue. The coordinate of each DNA triplet is calculated as the geometric center of the three corresponding nucleotides while the coordinate of Cβ atom of each residue represents the residue position (a pseudo Cβ is used for Glycine based on its geometric shape).

The calculation of VDW packing energy uses a harmonic form with soft repulsion and attraction terms as described previously [8]. Since the knowledge-based binding energy is derived from the mean force theory and, in principle, it covers all the energy effects including the VDW packing contribution. The primary role of adding this packing energy to the docking is to guide the docking process while not affecting the final docked structures (i.e. *W_packing _*approaches zero as the random walk progresses). The constraint energy is also used to guide the docking process by bringing the DNA molecule in contact with the protein surface. This energy becomes zero for correctly docked structures.

The simulated annealing sampling of the conformational space includes translation at a step size of 0.01Å and rotation at a step size of 2 degrees. The initial temperature is set to a 0.833 acceptance rate and the cooling rate is 0.998. The simulation continues until the system converges with an acceptance rate lower than 1% or when the total number of steps reaches a pre-set maximum (1.5 million in our current work). To improve coverage on the conformational search space, we conduct multiple Monte-Carlo simulations with different random seeds.

**Figure 2:**
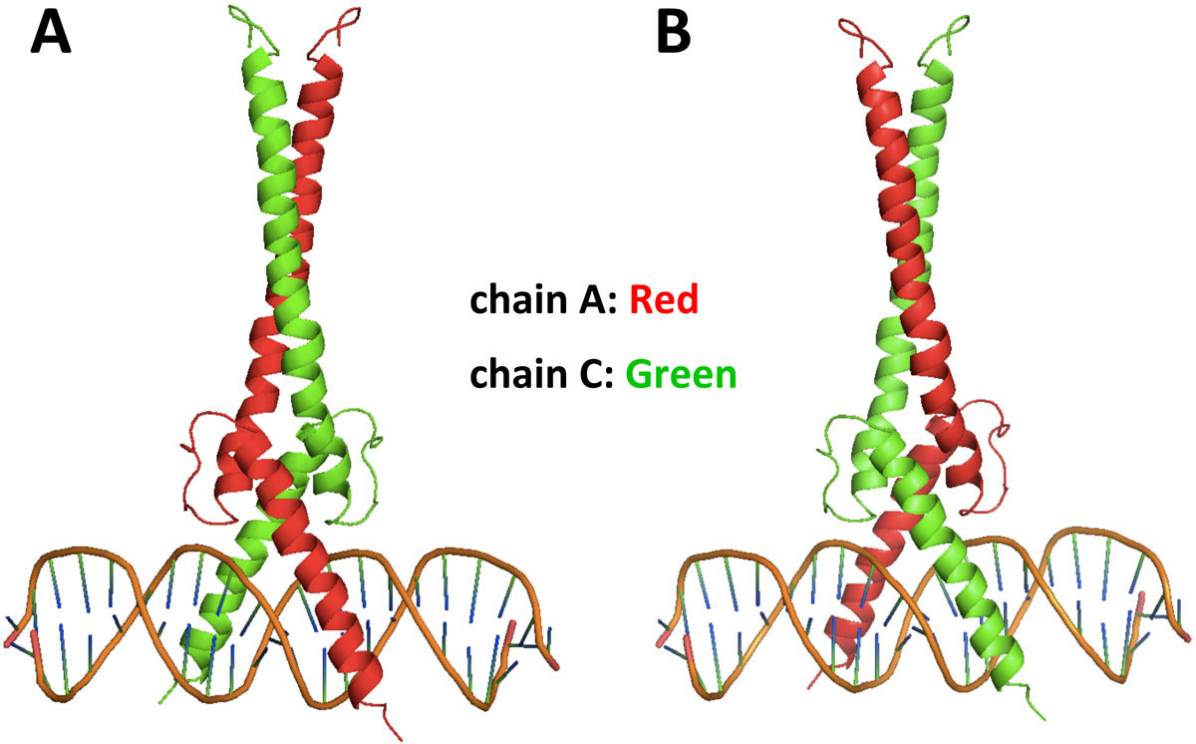
**Example of a homodimeric TF-DNA complex and flipped structures**. The complex structure of Max and its cognate DNA (PDBID:1AN2). Max recognizes its cognate DNA sequence through a homo-dimeric basic helix-loop-helix domain. A: native Max-DNA complex structure with chain A (red) and chain C (green); B: chain A and chain C are flipped.

To evaluate the docking performance, we compared the docked DNA conformations with the corresponding DNA structures in the native TF-DNA complexes by fixing the protein positions. The root mean square deviation (RMSD) is computed between the predicted and the native complex using DNA backbone heavy atoms. In some TF-DNA complexes, the proteins are homodimers that bind to the same or similar DNA sequences. In these cases, if the two protein chains exchange positions in the TF-DNA complex, the new structure should also be considered as "correct". For example, 1AN2 is a dimeric transcription factor Max that binds to its recognition sequence CACGTG by direct contact (Figure 2A) [22]. If chain A and chain C switch their positions, the newly generated structure is technically the same as the native TF-DNA complex (Figure 2B). To address this issue in docking automatically, we generated a "flipped" DNA structure by flipping the two protein chains. Then for docking evaluation, we calculate the RMSDs by comparing the docked structure with both the native and the "flipped" DNA structures and record the smaller RMSD for the docked TF-DNA complex.

### GPU computing

All the tests and experiments of our GPU algorithm were conducted on the 'Keeneland' GPU-based HPC system co-hosted by Georgia Institute of Technology, Oak Ridge National Lab, and the University of Tennessee [23]. The system currently contains 120 nodes, each equipped with 3 Nvidia M2070 GPU cards. Nodes in the system are connected via QDR infiniband switches. The software tools used were GCC 4.1, CUDA 3.2, and MPI 1.4.3-intel.

Our protein-DNA docking approach exhibits three levels of parallelism: (1) multiple protein-DNA pairs can be evaluated in parallel; (2) for each protein-DNA pair, multiple Monte-Carlo simulation runs are independent of each other; and (3) for each simulation run, the DNA movement and energy calculations feature fine-grained parallelism: the calculations are applied to each atom and amino acid/DNA triplet and can be performed in parallel within each simulation step. We design our GPU program to explore all three levels of parallelism. For notational convenience, we call each Monte-Carlo simulation run a task.

#### A. Task level design and scheduling

Our protein-DNA docking program is designed for high-performance computing (HPC) systems equipped with Nvidia GPU cards. Such platforms feature a 4-level hardware hierarchy: multiple computing nodes; each equipped with multiple Nvidia GPUs; each GPU contains several multiprocessors; and each of which consists of multiple processor cores. Such a hardware hierarchy maps to a 4-level software architecture: multiple MPI processes, each involving a GPU CUDA kernel, which contains multiple CUDA thread blocks. Each block consists of multiple CUDA threads. In this software architecture, the parallel tasks are mapped to different processes, simulation runs within each task are mapped to different CUDA thread blocks, and parallel operations inside each simulation step are mapped to different CUDA threads. In the design of our protein-DNA docking algorithm, the master process is responsible of dispatching tasks. The slave processes iteratively execute six operations: obtaining a task from the master, reading input data, copying data from CPU memory to GPU memory, executing the CUDA kernels, copying outputs from GPU memory to CPU memory, and writing outputs. We also include an additional pre-docking stage where the necessary information of the target protein-DNA pairs are retrieved from text files, assembled into appropriate data structures, and then stored as binary data files. With such pre-docking data ready, the master process can dispatch docking task by sending out a simple task ID, the slave process will load data file directly according to this ID. The MPI processes can thus be light-weighted, and the startup overhead can be significantly reduced.

#### B. CUDA kernel design

**Figure 3:**
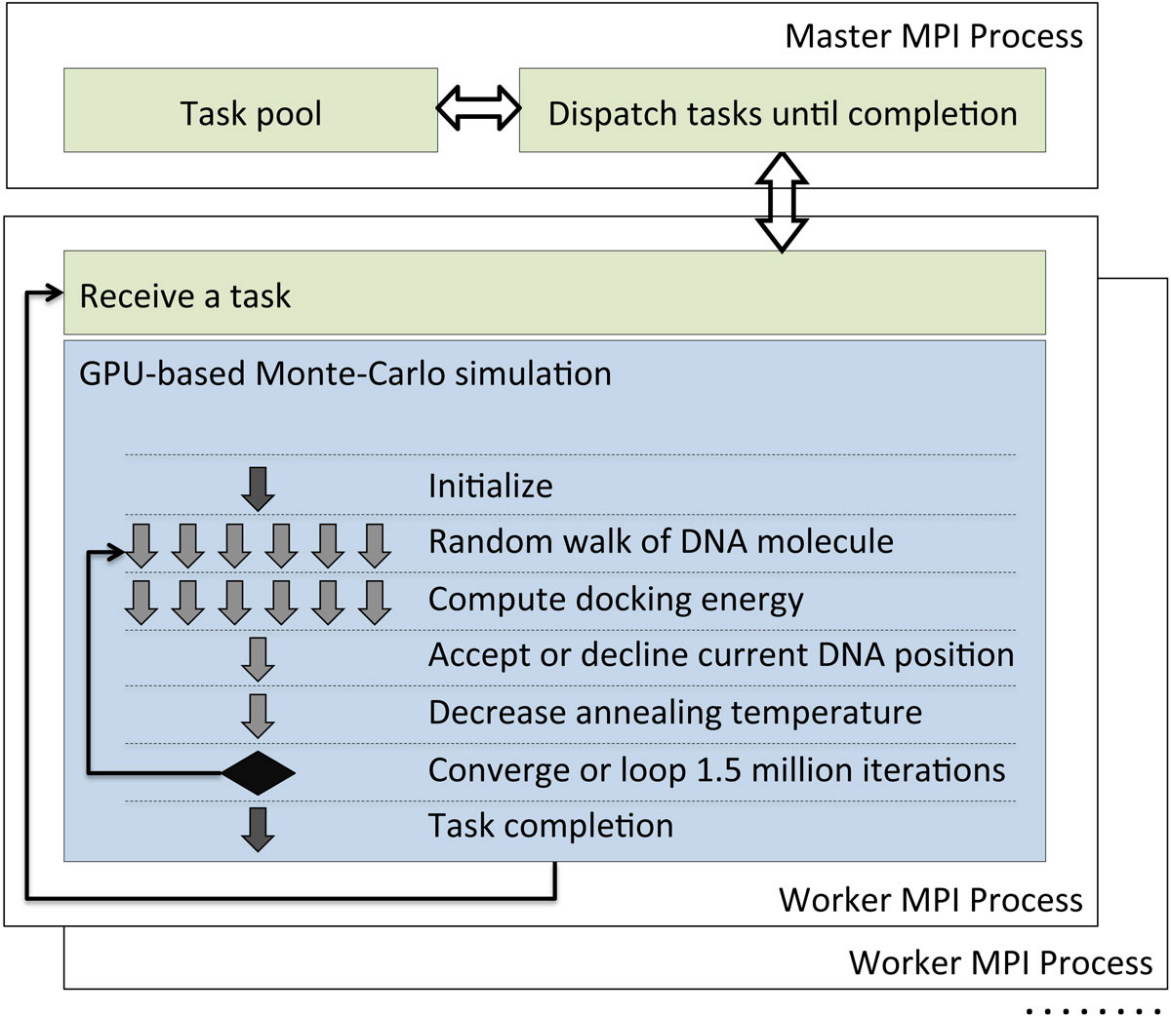
**Design of the docking algorithm at CUDA kernel level**.

The overall structure of the CUDA kernel for our Monte-Carlo simulated annealing based docking algorithm is illustrated in Figure 3. The memory spaces on GPU are pre-allocated to accommodate data structures for the TF-DNA complexes. Each time before the docking kernel is launched, the host MPI process loads a set of TF-DNA data into the GPU memory and runs a random number generation kernel to prepare the random numbers. Within the kernel, each block conducts an independent simulation run. The number of concurrent simulation runs is set equal to the number of multiprocessors available on the target GPU cards. For example, the number is 14 for Nvidia M2070 GPU cards. A reduction operation is used to compute the total interaction energy, and several small pieces of sequential code are used for setting parameters and making randomized simulated annealing decisions.

The computation of the VDW packing energy dominates the execution time. This interaction only affects atoms within close proximity and approaches zero as the distance increases. Consequently, in the computation, the entire 3D space is partitioned into small cells (6x6x6 Å), and for each DNA atom, we only evaluate its interaction energy with protein atoms in the same cell and in the neighboring 26 cells. Our algorithm uses 27 threads to evaluate one DNA atom with respect of its 27 neighboring cells (including the cell the atom resides). The binding affinity is evaluated between DNA triplets and protein residues. During the evaluations, the space is partitioned into lattices which are coarser-grained than cells for packing energy calculation. Since a molecule has fewer triplets/residues than atoms, the computation of binding affinity is faster than the packing energy.

## Results and discussion

### Computation cost of TF-DNA docking

**Figure 4:**
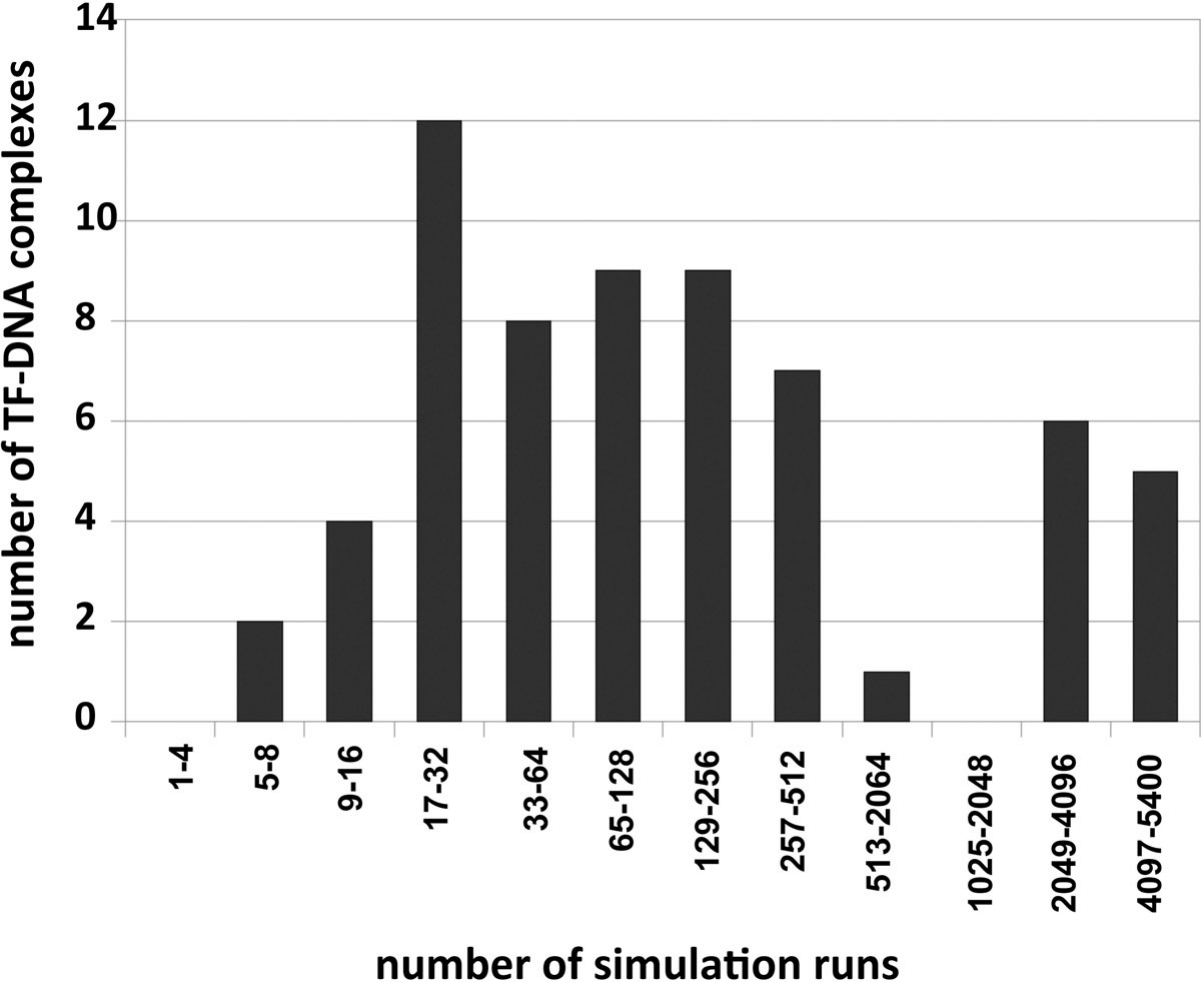
**The number of simulation runs to find at least one near-native conformation**.

The rugged docking energy landscape and the statistical nature of our docking algorithm suggest that an accurate conformation can be found only if sufficient Monte-Carlo simulations are conducted assuming the energy function is accurate. We first show the importance of conducting more simulation runs and the need for computational speedups. We conducted 5400 simulation runs for each complex in our dataset of the 75 TF-DNA complexes. Sixty-three out of the 75 complexes have at least one docked conformation with RMSD less than 2 Å. The number of runs needed to produce one near native conformation is summarized in Figure 4. It shows that the difficulty of conformational search varies for different TF-DNA complexes. For example, 12 out of the successfully docked complexes only need 17-32 simulation runs for the random search to 'hit' the near-native conformation. But there also exist 5 difficult complexes that need more than 4097 runs for one 'hit'. Since we do not know the search difficulty for a blind docking prediction, it is therefore critical to increase the number of Monte-Carlo simulation runs to improve the quality of the docked conformation.

### GPU-based TF-DNA docking efficiency

**Figure 5:**
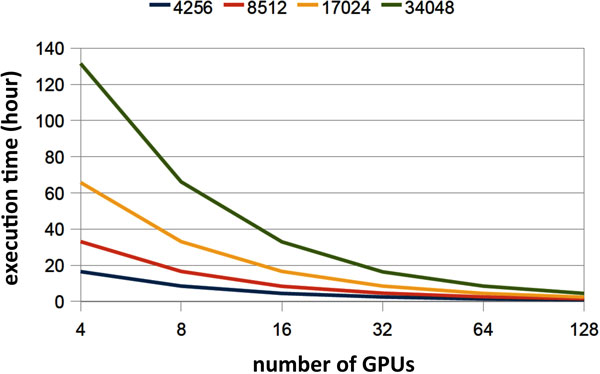
**Speed-ups of our GPU-based algorithm**.

We implemented our protein-DNA docking algorithm on GPU and tested the execution speed of our GPU docking program. We used a 2.8GHz Intel Xeon processor as the baseline to measure the speedup. Twenty Monte-Carlo simulation runs were conducted for each TF-DNA complex. The average speedup across all complexes is 28. A breakdown of the speedup shows that the calculation of the packing energy was accelerated by 40, and binding affinity by 35. Not surprisingly, speedups in moving the DNA and accepting new DNA positions are small due to the simplicity of these calculations. We also tested the scalability of our algorithm when executed on the GPU cluster. In the strong scaling experiment, we assigned 4256, 8512, 17024, and 34048 tasks to each GPU respectively. Figure 5 shows that our algorithm achieved close-to-linear speedups. When scaled to 128 GPU cards, our algorithm achieved a sustained speed of 10.4 TFLOPs, which is comparable to a traditional CPU cluster with 3600 processor cores.

### Improvement of TF-DNA prediction accuracy

To assess the improvement on prediction accuracy with our accelerated GPU algorithm, we ran the GPU docking algorithm with different number of Monte-Carlo simulations, 200, 400, 800, and 1600 using the data set of 75 TF-DNA complexes. The results are shown in Table 1 where a checkmark "x" indicates at least one conformation with RMSD < 1 Å was found during the search, and a checkmark in parenthesis "(x)" indicates a docking result where the conformation with the lowest energy has an RMSD less than 1Å. Our docking algorithm was successful in finding 47 of 75 (63%) near-native complexes when 200 Monte-Carlo runs were performed, and the sampling rate for finding near-native structures increased to 67%, 71%, and 73% when the number of Monte-Carlo runs increased to 400, 800, and 1600 respectively. When predictions were made based on the lowest energy, 36 (48%) complexes have been docked with RMSD < 1 Å with 200 Monte-Carlo runs. The success rate increased to 51%, 53%, and 55%, respectively as we increased the number of Monte-Carlo runs to 400, 800, and 1600. The above results demonstrated that increasing the coverage of the sampling space improves the chance of finding a near-native docking conformation, and our GPU based acceleration method is efficient in increasing the sampling coverage.

**Table 1:** Docking evaluation on 75 non-redundant TF-DNA complexes

		Conformation with the lowest RMSD		Conformation with the lowest energy		Number of Monte-Carlo runs			
**ID**	**PDB ID**	**E_docking_**	**RMSD(Å)**	**E_docking_**	**RMSD(Å)**	**200**	**400**	**800**	**1600**

1	1a02	2483.21	11.72	-87.22	22.51				
2	1a0a	-138.17	0.41	-140.53	0.46	x(x)	x(x)	x(x)	x(x)
3	1akh	-194.5	0.15	-195.44	0.17		x(x)	x(x)	x(x)
4	1am9	-246.58	0.1	-248.37	0.11	x(x)	x(x)	x(x)	x(x)
5	1an4	-50.06	8.05	-96.87	13.65				
6	1b72	-202.72	0.2	-203.79	0.4	x(x)	x(x)	x(x)	x(x)
7	1bdt	-335.33	0.08	-338.16	0.11	x(x)	x(x)	x(x)	x(x)
8	1cf7	-123.5	0.22	-125.81	0.37	x(x)	x(x)	x(x)	x(x)
9	1cma	-52.28	0.19	-56.19	20.75	x(x)	x(x)	x(x)	x(x)
10	1d3u	-390.32	0.21	-396.4	0.4	x(x)	x(x)	x(x)	x(x)
11	1d5y	-122.22	15.62	-178.55	63.19				
12	1dh3	-33.21	1.94	-38.8	30.76				
13	1efa	-171.99	0.06	-175.57	0.2	x(x)	x(x)	x(x)	x(x)
14	1g2d	-329.91	0.18	-336.89	0.64	x(x)	x(x)	x(x)	x(x)
15	1gd2	-192.36	0.08	-195.4	0.13	x(x)	x(x)	x(x)	x(x)
16	1gxp	-200.25	0.14	-205.16	0.22	x(x)	x(x)	x(x)	x(x)
17	1h6f	-195.97	0.09	-195.97	0.09				x(x)
18	1h88	-81.23	21.4	-112.61	70.87				
19	1h9d	-181.85	0.21	-185.43	0.28	x(x)	x(x)	x(x)	x(x)
20	1ic8	-358.99	0.17	-364.87	0.25	x(x)	x(x)	x(x)	x(x)
21	1je8	-30.9	6.09	-85.71	63.32				
22	1jfi	-320.51	0.21	-324.72	0.26	x(x)	x(x)	x(x)	x(x)
23	1jj4	-141.63	0.22	-143.72	0.4	x(x)	x(x)	x(x)	x(x)
24	1jt0	-275.84	0.09	-279.95	0.14	x(x)	x(x)	x(x)	x(x)
25	1ku7	-54.95	0.66	-72.58	22.43	x()	x()	x()	x()
26	1l3l	-93.27	3.49	-135.37	62.72				
27	1le5	-466.48	0.2	-470.46	0.22	x(x)	x(x)	x(x)	x(x)
28	1lq1	-232.93	0.38	-233.89	0.39	x(x)	x(x)	x(x)	x(x)
29	1mdm	-78.93	8.34	-87.35	50.2				
30	1mdy	-131.3	0.12	-133.91	0.23	x(x)	x(x)	x(x)	x(x)
31	1mnm	-232.86	0.1	-234.38	0.16	x(x)	x(x)	x(x)	x(x)
32	1nkp	-225.91	0.19	-226.15	0.19			x(x)	x(x)
33	1nlw	-260.52	0.43	-261.85	0.47	x(x)	x(x)	x(x)	x(x)
34	1ozj	-87.62	0.4	-89.39	0.53	x(x)	x(x)	x(x)	x(x)
35	1pp7	-41.26	3.34	-57.53	8.43				
36	1pue	-70.94	0.29	-80.75	56.96	x()	x()	x()	x()
37	1puf	-269.14	0.42	-271.22	0.5	x(x)	x(x)	x(x)	x(x)
38	1qne	-598.6	0.2	-606.45	0.36	x(x)	x(x)	x(x)	x(x)
39	1qp9	-220.62	0.17	-221.97	0.17	x(x)	x(x)	x(x)	x(x)
40	1r8d	-73.99	11.58	-106.13	35.66				
41	1rio	-179.09	0.08	-180.44	0.15	x(x)	x(x)	x(x)	x(x)
42	1rzr	-123.71	0.72	-142.16	37.7		x()	x()	x()
43	1skn	-32.36	4.35	-73.58	33.34				
44	1t2k	100.11	0.96	96.65	0.98	x(x)	x(x)	x(x)	x(x)
45	1ttu	-57.09	1.7	-78.09	30.8				
46	1u8b	-47.93	1.12	-83.26	30.23				
47	1u8r	595.89	0.76	262.38	98.73	x()	x()	x()	x()
48	1ysa	3800.94	12.51	-54.74	28.61				
49	1z9c	-160.1	0.21	-194.41	75.44	x()	x()	x()	x()
50	1zme	-38.31	12.31	-76.31	22.11				
51	1zrf	-155	0.25	-156.66	0.25	x(x)	x(x)	x(x)	x(x)
52	1zs4	-91.84	0.61	-104.68	46.32	x()	x()	x()	x()
53	2as5	-329	0.36	-340.64	0.46	x(x)	x(x)	x(x)	x(x)
54	2c9l	-38.93	0.48	-46.69	25.73				x()
55	2d5v	-193.42	0.42	-196.68	0.5	x(x)	x(x)	x(x)	x(x)
56	2drp	-303.41	0.16	-306.18	0.34	x(x)	x(x)	x(x)	x(x)
57	2er8	-23.48	0.16	-108.6	29.25	x	x()	x()	x()
58	2etw	-105.4	0.21	-106.11	0.26	x(x)	x(x)	x(x)	x(x)
59	2fio	-51.12	18.49	-146.84	46.95				
60	2gli	-194.58	0.18	-197.61	0.33	x(x)	x(x)	x(x)	x(x)
61	2h27	47.97	0.2	-86.91	23.1			x()	x()
62	2nll	-199.27	0.31	-201.17	0.51	x(x)	x(x)	x(x)	x(x)
63	2ntc	-50.65	3.26	-103.2	33.95				
64	2pi0	86.73	0.39	55.57	40.36	x()	x()	x()	x()
65	2ql2	-136.32	0.9	-140.33	1.02	x()	x()	x()	x()
66	2r1j	-262.07	0.27	-263.47	0.55	x(x)	x(x)	x(x)	x(x)
67	2w7n	-86.31	0.33	-91.12	34.7	x()	x()	x()	x()
68	2yvh	192.78	0.48	-46.35	54.44	x()	x()	x()	x()
69	3a5t	-91.62	0.18	-91.89	0.19		x(x)	x(x)	x(x)
70	3bs1	-51.78	5.92	-66.91	28.18				
71	3clc	-68.46	6.97	-94.63	12.53				
72	3coq	-30.5	18.87	-79.66	30.57				
73	3cro	-114.09	0.6	-119.95	1.07	x()	x()	x()	x()
74	3d1n	-944.87	0.19	-952.56	0.21	x(x)	x(x)	x(x)	x(x)
75	3dfx	-107.27	0.35	-107.27	0.35			x(x)	x(x)

Total						47(36)	50(38)	53(40)	55(41)

### Test on the rigid-body TF-DNA docking benchmark

We next tested our GPU accelerated sampling on the rigid TF-DNA docking benchmark [19]. The benchmark contains a carefully selected non-redundant set of 38 test cases, encompassing diverse fold families. The 38 test cases are classified into easy (21 cases) and hard (17 cases) groups with respect to the degrees of difficulty in TF-DNA docking. The benchmark was designed to identify the strengths and weaknesses of potential functions and docking algorithms and to facilitate the development of better approaches.

We ran our GPU docking simulations on this benchmark set with the number of simulation runs at 200, 800, and 1600 respectively. The detailed docking results are shown in Table 2, in which the first 21 entries above the line are the easy cases while the remaining cases are the hard ones. These docking results are summarized in Table 3. The results clearly show two different trends for the easy and hard targets. For the easy targets, increasing sampling runs from 200 to 1600 improved the number of successful predictions based on the lowest energy, from 7 to 9 within 1Å (or 8 to 10 within 3Å) when compared to the native TF-DNA structures. However, for the hard targets, more simulation runs did not improve the number of successful predictions with either 1Å or 3Å as a cutoff.

**Table 2:** Docking simulations on a rigid TF-DNA benchmark with 38 cases

PDB	200 simulation runs				800 simulation runs				1600 simulation runs			
	
	conformation with the lowest RMSD		conformation with the lowest energy		conformation with the lowest RMSD		conformation with the lowest energy		conformation with the lowest RMSD		conformation with the lowest energy	
	
	RMSD (Å)	E_docking_	RMSD (Å)	E_docking_	RMSD (Å)	E_docking_	RMSD (Å)	E_docking_	RMSD (Å)	E_docking_	RMSD (Å)	E_docking_
1aay	0.38	-208.30	0.42	-209.88	0.35	-208.39	0.44	-210.37	0.35	-208.39	0.44	-210.37
1an2	0.66	-119.22	0.66	-119.22	0.57	-118.31	0.66	-119.22	0.57	-118.31	0.66	-119.22
1jj4	0.39	-127.55	0.54	-129.26	0.38	-128.13	0.52	-129.42	0.30	-127.97	0.56	-129.73
1jt0	5.20	-82.27	5.20	-82.27	0.46	-124.44	0.46	-124.44	0.46	-124.44	0.46	-124.44
1lmb	0.16	-199.62	0.16	-199.62	0.07	-199.77	0.17	-200.04	0.06	-198.95	0.18	-200.19
1qn4	0.17	-313.59	0.40	-315.47	0.16	-312.87	0.41	-316.10	0.16	-312.87	0.39	-316.35
1qpi	4.47	-78.92	4.47	-78.92	4.47	-78.92	4.47	-78.92	3.42	-80.24	3.42	-80.24
1sax	1.12	14.81	28.63	-85.69	1.12	14.81	28.63	-85.69	1.09	16.15	19.15	-86.56
1tro	13.88	-57.73	16.76	-81.33	13.68	-60.80	20.82	-81.43	13.68	-60.81	20.84	-81.71
1z9c	1.02	-67.71	32.24	-124.23	1.02	-67.71	32.22	-124.68	0.98	-67.40	32.22	-124.68
1zs4	0.58	-99.50	30.17	-100.80	0.28	-95.76	30.18	-100.94	0.28	-95.76	0.69	-101.68
2ac0	0.22	125.31	14.40	103.80	0.22	125.31	14.40	103.15	0.21	123.26	14.45	103.06
2cgp	0.69	-162.09	0.83	-165.63	0.69	-162.60	0.83	-165.63	0.68	-162.92	0.83	-165.63
2e1c	4.08	-47.43	6.03	-65.54	1.03	-58.02	6.05	-65.72	1.03	-58.02	6.05	-65.72
2it0	10.10	-84.19	13.91	-88.21	10.10	-84.19	13.99	-88.93	7.02	-64.61	13.91	-89.00
2or1	1.05	-200.25	1.61	-209.88	0.90	-199.81	1.63	-210.06	0.86	-199.78	1.62	-210.64
2yvh	19.38	-79.05	36.64	-97.66	17.29	-67.95	35.64	-97.98	10.69	-62.60	35.64	-97.98
3clc	6.42	-61.80	20.43	-77.15	6.42	-61.80	20.43	-77.15	6.23	-51.19	20.43	-77.15
3dnv	0.58	-144.19	0.69	-145.67	0.58	-144.19	0.70	-145.68	0.58	-144.19	0.68	-145.71
3e6c	13.52	-68.30	16.25	-96.54	7.18	-61.11	16.22	-97.42	7.18	-61.11	16.22	-97.42
3gz6	3.68	-40.08	36.98	-43.99	2.34	-42.74	4.38	-44.12	2.34	-42.74	4.39	-44.18

1b01	0.95	4.05	10.57	-9.36	0.95	4.05	10.57	-9.36	0.95	4.05	10.61	-9.41
1by4	0.43	-186.88	0.43	-186.88	0.40	-185.19	0.42	-186.99	0.40	-185.19	0.42	-187.09
1cma	0.71	-46.26	0.71	-46.26	0.53	-33.93	0.71	-46.26	0.48	-33.24	0.71	-46.26
1gxp	1.21	-55.52	38.72	-74.32	0.93	-56.93	38.72	-74.73	0.93	-56.93	38.70	-74.80
1h8a	15.79	-106.71	18.70	-116.66	15.55	-108.11	18.70	-116.66	15.54	-108.31	18.73	-116.71
1hjc	2.00	-50.80	25.31	-67.89	2.00	-50.80	25.29	-68.16	2.00	-50.80	25.29	-68.16
1r8d	13.90	-57.78	35.96	-92.92	12.41	-82.12	35.92	-93.33	11.64	-67.42	36.12	-94.01
1rio	23.98	60.74	60.65	-43.07	23.95	61.05	60.65	-43.16	23.95	61.05	60.58	-43.44
1xpx	1.27	-56.51	20.96	-73.48	1.21	-56.52	20.96	-73.48	1.20	-56.85	20.96	-73.48
1zme	13.83	-44.24	32.10	-70.01	13.83	-44.24	32.09	-70.12	13.83	-44.24	32.12	-70.88
2bnw	3.47	-42.43	8.16	-44.98	3.41	-42.44	8.16	-44.98	3.40	-42.25	8.18	-45.05
2c6y	5.03	-56.01	31.52	-88.13	4.15	-58.13	31.52	-88.53	4.15	-58.13	31.52	-88.53
2fio	18.40	-52.54	43.65	-137.99	18.40	-52.54	33.45	-1245.70	17.68	-43.64	33.45	-1245.70
2irf	0.55	-88.97	0.96	-92.62	0.55	-88.97	0.95	-92.82	0.43	-87.69	0.96	-92.98
2rbf	6.35	-38.23	8.66	-52.78	6.35	-38.23	8.66	-52.78	6.35	-38.23	24.06	-53.50
2zhg	12.53	-45.59	28.78	-81.92	6.85	-54.71	28.76	-82.33	5.45	-50.31	28.75	-82.56
3hdd	4.19	-100.07	4.56	-102.31	4.09	-100.98	4.56	-102.31	4.09	-100.98	4.57	-102.40

**Table 3:** Summary of docking simulations on the rigid-docking benchmark

# of simulations	category	RMSD<1Å	RMSD<3Å
200	Easy	7(9)	8(12)
	
	Hard	3(4)	3(7)

800	Easy	8(11)	9(15)
	
	Hard	3(5)	3(7)

1600	Easy	9(12)	10(15)
	
	Hard	3(5)	3(7)

The docking simulations also produced some near-native structures ("conformation with the lowest RMSD" in Table 2 and the number in parenthesis in Table 3) that were missed due to the higher docking energies. More simulation runs resulted more near-native structures for the easy cases while there are almost no changes for the hard cases (Table 2). A larger percentage of the near-native structures in the easy cases also have the lowest energies than those in the hard cases. This is consistent with the rationale of assigning degrees of docking difficulty based on the interaction strength and suggesting that in addition to developing more efficient search algorithm we need better energy functions for discriminating the native or near-native structures from the decoy ones.

## Conclusions

Protein-DNA docking is a computation extensive problem. Due to the statistical nature of our Monte Carlo-based protein-DNA docking algorithm and the potentially rugged energy landscape, it is desirable to run multiple Monte-Carlo simulations with different seeds for better prediction performance. In this paper, we present a GPU-based high-performance protein-DNA docking algorithm that was designed for large scale GPU clusters. Modern GPU is not only a powerful graphics engine but also a highly parallel programmable processor featuring peak arithmetic and memory bandwidth that often substantially outpaces its CPU counterpart [17]. Rapid improvement in GPU programmability and capability has spawned a research community that has successfully mapped a broad range of computationally demanding problems to the GPU, including many bioinformatics and biomedical problems [24-27]. To take advantage of GPU's massive parallel processing capability, we developed this GPU-based docking algorithm to accelerate the random sampling process, and thus improve the performance of the TF-DNA docking algorithm. To this end, we designed special techniques to improve the efficiency of the CUDA kernel and the scalability over the entire cluster.

Experimental results using a non-redundant dataset demonstrated a 28x speedup using a single GPU card and close-to-linear scalability when using GPU clusters. Further testing on a rigid TF-DNA docking benchmark revealed that such improved computing capability improves the chance of finding near-native conformations for the easy cases in the benchmark, but not on the hard cases, suggesting there is a limit for improving prediction accuracy by simply increasing the number of simulation runs with our current energy function and search algorithm. There is clearly a need for developing more efficient search algorithms and more accurate interaction potentials.

## List of abbreviations used

CUDA: compute unified device architecture; GPU: graphical processing unit; HPC: high performance computing; MPI: message passing interface; NRBC: number of residue-base contact; PDB: protein data bank; RMSD: root mean square deviation; TF: transcription factor; VDW: van der Waals.

## Competing interests

The authors declare that they have no competing interests.

## Authors' contributions

BH and JTG conceived the project and wrote the manuscript. JW implemented the GPU docking program and performed the docking simulation and data analysis. TT prepared the datasets and generated the pre-docking data files. All authors read and approved the final manuscript.
